# What Human Concepts We Learn From Emergency Medicine Practice

**DOI:** 10.31661/gmj.v8i0.1553

**Published:** 2019-10-29

**Authors:** Maryam Massaeli, Masoud Shahabian

**Affiliations:** ^1^Emergency Medicine Department, AJA University of Medical Sciences, Tehran, Iran

**Keywords:** Emergency Medicine, Leadership, Patients

## Dear Editor,

This article aimed to provide the concept patterns obtained from the observations of a specialist in emergency medicine (EM) in the work setting. Some of these concepts in EM practice are expressed below.

### Leadership

Emergency physicians (EPs) are leaders of emergency departments (ED) who have to manage operational issues affecting the patients, crews, nurses, and wards as well as help bed management supervisor. As we know, the EDs are high-risk areas in healthcare settings. Therefore, the improvement of leadership skills and increase awareness of employing human factors lead to the enhancement of patient safety. The EPs should be qualified to function as a team leader and manager. Since these non-technical skills are not involved in the medical courses, learning non-clinical skills are necessary for reducing the risk of errors [1].

### The Importance of Rapid Decision Making When Seconds Count

In contrast with other medical fields, ED diagnostic and therapeutic decisions should be reached in a matter of seconds or minutes. The EPs has just a few minutes to gather the multidisciplinary hospital team, deal with the confusing situations, manage the team’s efforts, and decide the right treatment for each patient. Therefore, seconds are critical, and it is the advantage of this field which is not present in other medical courses. The EP can save patients’ lives in just a few seconds. Correct decision-making is among the skills each EP should acquire. There are several treatment options with a range of different possible outcomes for each patient. A misleading choice may have irreparable consequences because the death and life of the patient is the issue [2].

### A Judge in ED

The EPs are considered judges. They should act impartially regarding the choice and the type of treatment approach. The EPs should react based on the professional principles and must be able to resolve the conflict instantly; therefore, it is possible to use the ultimate emergency capacity [3]. In case of conflicts, the EP make judgments of patients and the medical system. Moreover, it is very important that an EP be able to handle the conflicts between two diseases, companions with their patients, specialists, or nurses, the patients with specialists and nurses, and specialists and nurses with each other.

### Each Patient Has a Story

The entrance to ED means an important challenge in patent life. All patients have interesting stories to tell before and after the treatment in the ED. These stories are mixed with patients’ social life, and they expect their stories to be heard. According to the literature, there is a positive relationship between physicians’ empathy and patients’ clinical outcomes, which introduces physicians’ empathy as a necessary factor regarding patient outcomes [4].

### The Importance of Team Working

In critical settings, it stands to reason that interprofessional teamwork is important; however, it is not fully conceptualized in practice. Working together and collaboration with different therapeutic groups is among the main issues in EM. A collection of defined features related to team, context, and goal are affiliated to teamwork in an emergency condition; therefore, we should attempt to improve these attributes [5]. The lack of presence of any treatment members may lead to a problem in the treatment of patients.

## Conflict of Interest

The authors have no conflict of interest.
